# Continuance compliance of privacy policy of electronic medical records: the roles of both motivation and habit

**DOI:** 10.1186/s12911-018-0722-7

**Published:** 2018-12-18

**Authors:** Kuang Ming Kuo, Yu Chang Chen, Paul C. Talley, Chi Hsien Huang

**Affiliations:** 1 Department of Healthcare Administration, I-Shou University, No.8, Yida Rd., Yanchao District, Kaohsiung City, 82445 Taiwan, Republic of China; 2 Department of Family Medicine, E-Da Hospital, Kaohsiung City, Taiwan, Republic of China; 3 Department of Applied English, I-Shou University, No. 1, Sec. 1, Syuecheng Rd., Dashu District, Kaohsiung City, 84001 Taiwan, Republic of China; 4 Department of Community Healthcare & Geriatrics, Nagoya University Graduate School of Medicine, Nagoya, Japan; 5 Center for Evidence-based Medicine, E-Da Hospital, Kaohsiung City, Taiwan, Republic of China; 6 School of Medicine for International Students, I-Shou University, Kaohsiung City, Taiwan, Republic of China

**Keywords:** Continuance compliance intention, Electronic medical records, Habit, Motivation, Privacy policy, Compliance behaviors

## Abstract

**Background:**

Hospitals have increasingly realized that wholesale adoption of electronic medical records (EMR) may introduce differential tangible/intangible benefits to them, including improved quality-of-care, reduced medical errors, reduced costs, and allowable instant access to relevant patient information by healthcare professionals without the limitations of time/space. However, an increased reliance on EMR has also led to a corresponding increase in the negative impact exerted via EMR breaches possibly leading to unexpected damage for both hospitals and patients. This study investigated the possible antecedents that will influence hospital employees’ continuance compliance with privacy policy of Electronic Medical Records (EMR). This is done from both motivational and habitual perspectives; specifically, we investigated the mediating role of habit between motivation and continuance compliance intention with EMR privacy policy.

**Methods:**

Data was collected from a large Taiwanese medical center by means of survey methodology. A total of 312 responses comprised of various groups of healthcare professionals was collected and analyzed via structural equation modeling.

**Results:**

The results demonstrated that self-efficacy, perceived usefulness, and facilitating conditions may significantly predict hospital employees’ compliance habit formation, whereas habit may significantly predict hospital employees’ intention to continuance adherence to EMR privacy policy. Further, habit partially mediates the relationships between self-efficacy, perceived usefulness, facilitating conditions and continuance adherence intention.

**Conclusions:**

Based on our findings, the study suggests that healthcare facilities should take measures to promote their employees’ habitualization with continuous efforts to protect EMR privacy parameters. Plausible strategies include improving employees’ levels of self-efficacy, publicizing the effectiveness of on-going privacy policy, and creating a positive habit-conducive environment leading to continued compliance behaviors.

## Background

It has been duly acknowledged that the adoption of electronic medical records (EMR), a collection of software functions commonly utilized in the delivery of directives for patient care, in the maintenance of patients’ medical records, and the distribution of laboratory testing or radiologic examinations results [1], can improve healthcare quality and decrease overall cost [2]. Via EMR, healthcare professionals can serve to inquire after patient information immediately without the limitations of time and space [3]. However, with the advent of a more accessible and comprehensive EMR system, a massive amount of medical records may become easily obtainable to both unauthorized and authorized users who are both inside and outside the healthcare facilities [4]. EMR are therefore potentially susceptible to security breaches which may lead to real patients’ privacy concerns [1]. Most reported privacy violations in healthcare facilities stem in fact from staff misuse or abuse of their privileged access status to patient records [5, 6].

There is a widely accepted knowledge as to the importance of employees’ compliance with organizational rules, procedures, and policies, all of which can be used to regulate employees’ accurate attitudes or behaviors regarding how organizational resources should be utilized [5, 7, 8]. Despite such evidence, employees often demonstrate not to abide by such prescriptive rules or policies. In reality, non-compliance to such stated policies may cause organizations significant reputational damage, remediation costs, or even subsequent penalties [9]. For many healthcare facilities, regulations have been purposefully mandated in order to secure patient information due to the increased digitization of patient records [10]. Healthcare facilities must therefore invest in how to effectively motivate employees to comply with stated policy and to secure EMR.

Although existing literature has significantly enhanced our understanding of the drivers for privacy policy compliance [4, 5, 8, 11], little is known about what happens afterwards. Persuading hospital employees to adhere to EMR privacy policy is of limited value if they subsequently eschew it and then return to risky non-compliance behaviors. One of the distinct aspects relating to the healthcare context is that the leaking of patients’ confidential medical information may cause greater risks than they may in other contexts [2]. Hence, to better understand what drives repeated compliance behavior, the incidence of previous adherence behavior may be inadequate, leaving a need to develop a more relevant model to find out those driven salient factors.

Research on repeated behavior indicates that both motivation and habit can play a role in continued compliance behavior [12–15]. Motivation has been identified as a key driver of general behavior [16], health behavior [17], and compliance behavior [13, 14]. Further, promoting habits requires a prior knowledge of the habit-formation process, but a paucity of privacy policy-compliance related habit formation literature exists since few studies have adequately investigated the relationship between motivation and habit [17].

Our aims in this study are therefore, based on both the motivational and habitual perspectives, to investigate the following research questions: (1) Does habit predict continuous EMR privacy policy compliance intention? And, (2) What are the motivational antecedents of habitual compliance? Knowledge about the impact and antecedents of habit is not only helpful in knowing how and why privacy compliance habits are formed, but it may also provide valuable guidelines on how to better develop hospital staff’s habit of continuance adherence to privacy policy in most cases.

### Theoretical foundation and hypotheses

The theoretical foundation of our study is adapted from self-determination theory [18] and habit-related perspective [19]. Self-determination theory, one of the often-adopted theoretical frameworks within motivation research [20], differentiates between various types of motivation according to the different reasons or goals leading to a given action [18]. Among those motivations, self-determinant theory differentiates between two main types of motivation: (1) intrinsic motivation, and (2) extrinsic motivation [18]. Intrinsic motivation refers to undertaking a behavior because it is inherently enjoyable or interesting to do, while extrinsic motivation refers to doing something because it can result in a separable outcome [16]. Such intrinsic and extrinsic motivation when taken together affect an individual to undertake a particular behavior [21].

Habits are commonly considered to be “*learned sequences of acts that become automatic responses to specific situations, which may be functional in obtaining certain goals or end states* [19] (p.14).” Many behaviors that are of interest to individuals, especially when they are repeatedly and satisfactorily executed, may eventually become personal habits without extra cognitive processes having to take place [19]. Although some researchers hold that habits can be formed quickly, most studies argue that habit continuation requires a certain amount of repetition or practice to take place [22, 23]. As for the formation of habits, literature [24] suggests that a stable context which requires an individual’s minimal attention in responding to certain situations, is essential. Once a habit is shaped, an individual can then perform a behavior automatically [25].

By integration of the motivation perspective from the standpoint of self-determination theory and the habit perspective, we argued that full protection of EMR privacy can be achieved via hospital staff’s habitual compliance behavior. This is due in large part because significant amounts of patient information is accessible through even a single breach if hospital staff are habitually in non-compliance with stated privacy policy. Further, enhancement of the hospital staff’s motivation to act responsibly and accordingly is also required [26] so that both intrinsic and extrinsic motivation, based on self-determination theory, can thus be assumed as helpful in forming the hospital staff’s policy compliance intention.

**Fig. 1:**
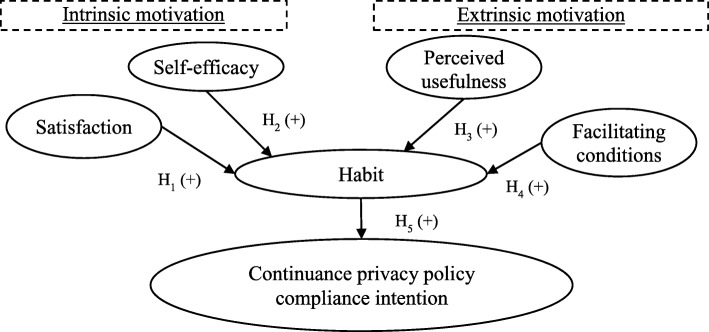
Research model and hypotheses

Figure 1 depicts our proposed research model. The model proposes that both intrinsic motivation and extrinsic motivation can serve to motivate the hospital staff’s habit formation for adherence to EMR privacy policy. Intrinsic motivation includes satisfaction (referring to hospital staff’s feelings related to prior compliance experiences with stated privacy policy) and self-efficacy (referring to the extent to which hospital staff’s assurance of adherence to stated privacy policy is measured). Extrinsic motivation includes perceived and measurable usefulness (referring to the hospital staff’s perceptions of the benefits of adherence to stated privacy policy) and facilitating conditions (referring to the extent to which the hospital staff believes that resources such as EMR, related software, hardware, and procedures exist to support their compliance to stated privacy policy). If the hospital staff’s habits (measuring the degree to which hospital staff tends to regularly abide with stated privacy policy because of learning attended from prior policy compliance experience) regarding adherence to EMR privacy policy are formed, they are then expected to have continuous compliance intention of EMR privacy policy. Each construct investigated in our research model and hypotheses is discussed below.

### The relationship between satisfaction and habit

Prior satisfaction experiences are an important condition for habit development as they will guide an individual’s dispositions to repeat the same action in order to attain his/her goal [22]. Therefore, if one has achieved his/her intended goals by undertaking a particular behavior and the experience turns out to be satisfactory, a repetition of the same action is more than likely [22, 27]. The higher the frequency, the stronger the habit [25]. Hospital staff members are requested to comply with a stated privacy policy when they become employed in hospitals, meaning they have the prior experience of adherence to privacy policy. If such an experience is pleasant, they are more likely to adhere to stated privacy policy repeatedly, which is necessary for developing a compliance habit. Evidence [23, 28, 29] supports this notion stating that satisfaction is positively linked with habit formation. It therefore follows that:

- **H**_**1**_**:** A hospital staff’s level of satisfaction with prior compliance to privacy policy is positively associated with his/her personal habit formation.

### The relationship between self-efficacy and habit

Literature has regarded habit as an automatic behavioral tendency [30] and many actions occur without cognitive efforts [31]. Generally speaking, since an individual’s effort and time are limited resources, he/she may thus allocate such finite resources to differing activities. It may imply that one activity may be perceived as being easier to perform than another and is therefore more likely to be conducted by the individual. In the context of our study, if hospital staff possess a higher level of self-efficacy, it will be easier for him/her to comply with stated privacy policy. And, we expect such a high self-efficacy will finally result in the formation of his/her habit of repetitive adherence to privacy policy. A number of studies [32–35] have found a relationship between self-efficacy and policy compliance intention or continuance intention to protect security. Further, research on the role of self-efficacy [29] has suggested the importance of self-efficacy on forming habits. We therefore propose the following:

- **H**_**2**_**:** Self-efficacy will have a positive relationship with the formation of a hospital staff’s habit to continue compliance with stated privacy policy.

### The relationship between perceived usefulness and habit

Davis [36] found that perceived usefulness significantly associated with individuals’ usage of information technology since users perceive that such utility will help them to attain the desired performance or goals they seek. The higher the perceived usefulness of compliance with stated privacy policy by hospital staff, the more likely the staff will take the same actions more often [22]. Associated feelings of achieving effective protection on patient privacy may then contribute to an increased level of perceived usefulness as the compliance behavior is performed frequently. Eventually, and as a result of simple repetitive behaviors, hospital staff may develop the habit of automatic adherence to stated privacy policy. Based on this argument, perceived usefulness could be an important antecedent in developing a hospital staff’s habit for adherence to stated privacy policy. Recent evidence suggests that the perceived security protection mechanism or perceived effectiveness, concepts that are akin to perceived usefulness of privacy policy in our study, are associated with policy compliance intention [37, 38]. Further, it is known that prior studies have suggested the relationship between perceived usefulness and habit [39, 40]. We therefore hypothesize the following:

- **H**_**3**_**:** Perceived usefulness is positively associated with hospital staff’s habit of adherence to stated privacy policy.

### The relationship between facilitating conditions and habit

Triandis [41] argued that behavior cannot happen if objective conditions in the environment are to discourage it. The purpose of these supporting resources is to remove any of the potential barriers hospital staff may face when they act according to privacy policy and to assist hospital staff in achieving the intended goal of protecting patients’ privacy. If facilitating conditions do not permit hospital staff to comply with privacy policy, they are unable to do so even if they perform the behavior habitually. With sufficient facilitating conditions present, adherence to stated privacy policy may thus likely become a matter of automatic responses from hospital staff since facilitating conditions provide for a stable context which can promote their habit formation [23]. Prior studies usually investigated the association between facilitating conditions and behavioral intention [15]. However, to the best of our knowledge, little attention has been paid to the examination of the relationship between facilitating conditions and the formation of habit. We expect the following hypothesis:

- **H**_**4**_**:** Facilitating conditions will have a positive relationship with the formation of a hospital staff’s habit to continuously compliance with stated privacy policy.

### The relationship between habit and continuous privacy policy compliance intention

Habit-related research [15, 23, 29, 42] suggests that many actions performed by individuals occur without a conscious decision to act, and they are engaged in because individuals are simply used to performing them on a repetitive or continuous basis. In other words, these repeated behaviors are habitual [41]. Previous studies [28, 43, 44] have reported the link between habit and behavioral intention. Transferring the rationale from prior habitual studies to our given context, if prior experience of compliance with stated privacy policy becomes habitual in nature, hospital staff members will be more likely to adhere to some or all of the stated privacy policy in an unthinking, lock-step or rote manner. In light of the above expectations, we hypothesize as follows:

- **H**_**5**_**:** Hospital staff’s continuous intention to gain adherence with stated privacy policy is positively associated with their habits regarding compliance with privacy policy.

## Methods

### Setting

In Taiwan, hospitals are usually divided into three major categories based on given characteristics of size: medical centers, regional hospitals, and district hospitals. Totally, the number of medical centers, regional hospitals, and district hospitals in Taiwan are about 19, 80, and 308, respectively [45]. Most of the three types of hospitals have utilized some form of EMR due to efforts to improve EMR adoption promoted by the Ministry of Health and Welfare in Taiwan. Medical centers, however, implement more comprehensive EMR systematization and have wider application of EMR than regional and district hospitals due to appreciable organizational resources. We therefore surveyed hospital staff from a medical center of 1300-beds serving nearly 5000 outpatients daily located in southern Taiwan. The subject hospital has about 3511 employees including 3020 healthcare professionals and 491 administrative staff. The subject hospital was chosen because of two major considerations: (1) The subject hospital is equipped with well-established EMRs systems aimed at providing patients with high quality healthcare services; and, (2) The subject hospital was regarded as being rather proactive in their use of EMR in terms of overall internal EMR utilization and the amount of EMR exchanged with external healthcare facilities [46]. Both reasons made the chosen hospital suitable for use in the study of EMR privacy protecting issues.

### Design

Our study used a cross-sectional design, and survey methodology was adopted to collect data. Since most violations of patient privacy in healthcare facilities stem from staff misuse or abuse of the privileged right to access patient records [5, 6], hospital employees (i.e., healthcare professionals and administrative staff) who are granted access EMR must still be regarded as potential threats capable of jeopardizing EMR privacy. Among 3511 employees involved as part of this study, about 2800 healthcare professionals and 100 administrative staff were actually authorized to access EMR. Considering the heavy workload of hospital staff, a census of all eligible employees is unfeasible, we therefore adopted convenience sampling to collect relevant data. We appointed a coordinator for the departments whose staff members maintain access to EMR systems in order to assist questionnaire administration. Recruitment of hospital employees was voluntarily and guaranteed anonymity to participate in the survey. Ethical approval from the subject hospital was sought and then acquired prior to the eventual administration of the survey.

### Measures

Our study used paper-and-pencil questionnaires for purposes of data collection. Following Churchill’s [47] suggestions for scale development, we adapted questionnaire items from existing validated scales in the extant literature in order to establish an initial item pool for each construct. Since adapted items were not originally designed for use in a healthcare context, we modified subsequent items to fit within an EMR setting. For example, an item for measuring perceived usefulness was, ‘Overall, I find the electronic mail system useful in my job.’ We changed this item to read, ‘Overall, compliance with EMR privacy policy is advantageous.’ An expert panel, consisting of one professor specializing in healthcare information management and two experienced EMR experts, reviewed postulated items to appraise their content validity. A few equivocal words were modified per the recommendations proposed by the panel in order to remove any possible misunderstandings during survey administration. In this questionnaire, all the items, with the exception of items pertaining to demographic information, were measured using a seven-point Likert scale, ranging from 1, representing “strongly disagree,” to 7, representing “strongly agree.” Composite reliability and Cronbach’s α were used to assess the reliability while discriminant validity and convergent validity were adopted to evaluate validity [48, 49].

The instrument for satisfaction was measured using four items adapted from [50]; whereas, the measurement scale for self-efficacy was measured using four items adapted from Taylor and Todd [51]. Perceived usefulness and facilitating conditions were measured using three items and four items adapted from Davis [36] and Taylor and Todd [51], respectively. Habit was measured by three items adapted from Limayem and Cheung [52]. Continuance intention to comply with privacy policy was measured with three items adapted from Bhattacherjee [50]. A pilot test was then conducted on 20 hospital staff members. Further modification to words and phrasing was made to the suggested items, leading to a final questionnaire justified for further testing. Appendix shows the final survey items as it was used in our study.

### Statistical analysis

Partial least squares (PLS), a distribution-free analytic method [49], was used for purposes of data analysis because the collected data did not follow a normal distribution (*p* < .001) to some extent subsequent to a Kolmogorov-Smirnov test. We utilized R software version 3.5.1 [53], with both plspm version 0.4.9 and semPLS version 1.0–10 package [54, 55], to assess the measurement model (i.e., dealing with the relationships between the observed variables and the latent variables) and the structural model (i.e., dealing with the relationships between the exogenous and endogenous variables) of PLS, respectively. In order to obtain the scores of the latent variables for subsequent use of measurement and in the structural model, their associated observed variables are weighted and summed by PLS [55]. Further, the RMediation version 1.1.4 [56] package was used to assess the mediation effect of habit.

## Results

### Respondent characteristics

Among the 2900 eligible hospital employees from differing participative units, we dispensed 350 questionnaires to those units that were willing to take part in our survey. From January 1st to January 31st, 2016, totally, 320 responses were collected, showing a response rate of 93.33%. Excluding eight unusable responses due to the presence of incomplete answers, 312 useful responses remained for subsequent analysis. Of the 312 valid responses, 56.78% of the given responses were from female respondents and 43.27% were from males. Nearly 75% of the respondents were aged 30–49 years old. Further, most of the respondents were university- or graduate school-educated (94.87%). Physicians and administrative staff together comprised the largest group of participants (51.92%), and over 58.33% of respondents claimed of having more than 10 years of healthcare-related work experience. Further, all respondents reported to understand the connation of EMR privacy policy, with 36.54% of respondents being not very sure about the particular contents of such stated policy in their facility. Respondents’ demographic information is provided in Table 1.

**Table 1:** Respondent characteristics

Category	Item	Healthcare professional (*N* = 230)		Administrative staff (*N* = 82)	
		Frequency	Percentage	Frequency	Percentage
Gender	Male	99	43.04	36	43.90
	Female	131	56.96	46	56.10
Age	20–29	52	22.61	5	6.10
	30–39	95	41.30	41	50.00
	40–49	68	29.57	31	37.80
	46–65	15	6.52	5	6.10
Education	High school	1	0.43	3	3.66
	College	10	4.35	2	2.44
	University	164	71.30	48	58.54
	Graduate school	55	23.91	29	35.37
Working experiences (years)	1–3	39	16.96	9	10.98
	4–6	44	19.13	11	13.41
	7–9	19	8.26	8	9.76
	≧10	128	55.65	54	65.85
Knowing privacy policy	Aware and fully understand	125	54.35	73	89.02
	Aware but not know all of it	105	45.65	9	10.98

Note: Some numbers in this report may not add up due to rounding effect

### Measurement model assessment

In PLS, the measurement model assesses the reliability and the validity of measures taken [48, 49]. Literature suggests reliability can be evaluated via composite reliability or Cronbach’s α [49]. In our study, both the values of composite reliability and Cronbach’s α of all constructs (see Table 2) were larger than the suggested value of 0.7 [49], thus demonstrating sufficient reliability being present.

**Table 2:** Descriptive statistics, reliability, and validity of constructs

Constructs (Code)	*M*	*SD*	Cronbach’s α	Composite reliability	Average variance extracted	Correlations among constructs					
						A	B	C	D	E	F
Self-efficacy (A)	5.22	0.84	0.94	0.92	0.81	**0.90**					
Facilitating condition (B)	5.14	0.85	0.90	0.82	0.74	0.76	**0.86**				
Satisfaction (C)	5.23	0.85	0.97	0.95	0.88	0.79	0.77	**0.94**			
Perceived usefulness (D)	5.33	0.83	0.95	0.92	0.87	0.70	0.67	0.84	**0.93**		
Habit (E)	5.33	0.83	0.95	0.92	0.87	0.78	0.81	0.78	0.74	**0.94**	
continuous privacy policy compliance intention (F)	5.36	0.84	0.95	0.93	0.87	0.76	0.77	0.76	0.73	0.84	**0.91**

Note:

*M* mean, *SD* standard deviation

Diagonal bold elements show the square root of average variance extracted

As for validity, convergent validity and discriminant validity are commonly assessed in terms of PLS [49]. Table 3 shows that all items in our study loaded highly on the postulated factors and had factor loadings greater than 0.7 [49]. Regarding the validity of construct level, the constructs used in this study had a value of average variance extracted higher than 0.5 [48], indicating sufficient convergent validity (see Table 2). Further, the squared root of average variance extracted for each construct was larger than the correlation coefficients of the specific construct with any other constructs in the proposed model, also demonstrating adequate discriminant validity [48].

**Table 3:** Factor loadings of constructs

Construct	Number of items	Loadings
Self-efficacy	4	0.89–0.91
Facilitating condition	3	0.75–0.92
Satisfaction	3	0.92–0.95
Perceived usefulness	3	0.91–0.94
Habit	3	0.91–0.96
Continuous privacy policy compliance intention	3	0.81–0.96

Several correlations between constructs are higher than 0.7 (see Table 2), but they are still lower than 0.85, which may indicate the presence of a collinearity issue [57]. To avoid the possible influence of collinearity, we further checked for the issue and the results demonstrated that the tolerance value of each construct investigated ranges from 0.15–0.64, showing that collinearity should not become an issue in this study [49].

### Structural model assessment

**Fig. 2:**
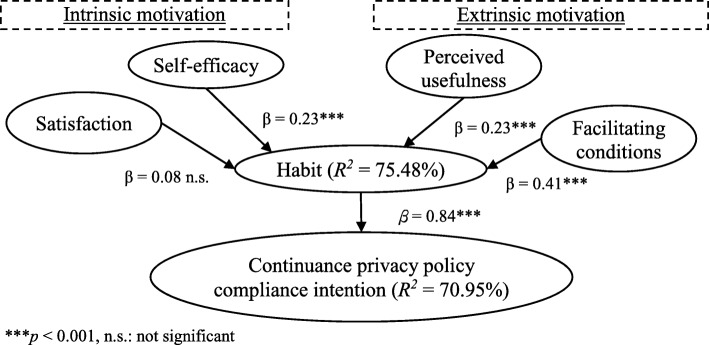
Structural model results

After assessing the measurement model, we then validated the hypotheses by inspecting the structural model. A bootstrapping procedure was adopted to assess the statistical significance of each path coefficient. Figure 2 demonstrated the structural model results. The five postulated hypotheses were all supported with correct signs, with the exception being Hypothesis 1. Contrary to our expectation that satisfaction did not predict habit, Hypothesis 1 was not supported (*p* = 0.193). Hypothesis 2 suggests a positive association of self-efficacy with habit, and we find support for this association (β = 0.23, *p* < .001). Hypothesis 3 posits that perceived usefulness significantly associates with habit. This was confirmed with a significant and a positive path coefficient (β = 0.23, *p* < .001). Regarding the role of facilitating conditions, Hypothesis 4 posits a positive relation between facilitating conditions and habit. The path coefficient (β = 0.41, *p* < .001) from facilitating conditions to habit was significant and positive, supporting Hypothesis 4. Finally, Hypothesis 5 posits that habit predicts continuance intention to comply with privacy policy. The path coefficient from habit to continuous intention was significant and positive (β = 0.84, *p* < .001), thus Hypothesis 5 was supported. Overall, about 75.48 and 70.95% of the variance of the habit and behavioral intention to continuous compliance with privacy policy can be accounted by the research model. Table 4 presents the structural model results with path coefficient, *t* value, and *p* value.

**Table 4:** Structural model results

Hypothesis		Path coefficient	*t* value	*p* value	Results
H_1_	Satisfaction➔Habit	0.08	1.30	0.193	Failed to support
H_2_	Self-efficacy➔Habit	0.23	4.62	< 0.001	Supported
H_3_	Perceived usefulness➔Habit	0.23	4.51	< 0.001	Supported
H_4_	Facilitating condition➔Habit	0.41	8.55	< 0.001	Supported
H_5_	Habit➔Continuous privacy policy compliance intention	0.84	27.52	< 0.001	Supported

In addition to testing the proposed hypotheses, we further examined the PLS structural model with three widely adopted criteria, namely the predictive relevance *Q*^*2*^, the *q*^*2*^ and the *f*^*2*^ effect size [49]. The *Q*^*2*^ value of habit was 0.64, showing the structural model had predictive relevance for this construct [49]. Further, the relative impact of predictive relevance (*q*^*2*^) of satisfaction, self-efficacy, perceived usefulness, and facilitating conditions was − 0.015, 0.042, 0.038, and 0.128, respectively. These *q*^*2*^ sizes were deemed small according the criteria suggested by the literature [49]. Finally, the exogenous constructs of satisfaction, self-efficacy, perceived usefulness, and facilitating conditions for explaining the endogenous construct habit have *f*^*2*^ effect sizes of 0.005, 0.070, 0.238, and 0.066, respectively. According to their effect sizes, these facilitating conditions had a medium effect size (*f*^*2*^ = 0.238), as well as possessing small predictive relevance (*q*^*2*^ = 0.128) [49]. Both self-efficacy and perceived usefulness were seen to have a small effect size and a small amount of predictive relevance. Satisfaction had the smallest effect size and also the smallest amount of predictive relevance in our study.

### Assessment of the mediation effect of habit

We followed suggested methodology steps [56] for establishing the mediation effect of habit. As shown in Table 5, habit was seen to significantly mediate among all the relationships besides the suggested relationship between satisfaction and continuance intention. The type of mediation found is a form of partial mediation for the relationships between self-efficacy, facilitating conditions, perceived usefulness and continuous intention to comply with privacy policy (see Table 5).

**Table 5:** Results of mediation analysis

Path	Direct effect	SE	Total effect	*SE*	Mediation type
Self-efficacy➔Habit	0.234***	0.05			
Facilitating condition➔Habit	0.412***	0.05			
Satisfaction➔Habit	0.082	0.06			
Perceived usefulness➔Habit	0.233***	0.05			
Self-efficacy➔Habit➔Continuous privacy policy compliance intention			0.197**	0.043	Partial mediation
Facilitation condition➔Habit ➔ Continuous privacy policy compliance intention			0.347**	0.042	Partial mediation
Satisfaction➔Habit ➔ Continuous privacy policy compliance intention			0.070	0.041	No mediation
Perceived usefulness➔Habit ➔ Continuous privacy policy compliance intention			0.197**	0.041	Partial mediation
Habit➔ Continuous privacy policy compliance intention	0.84***	0.06			

Note:

*SE* standard error

**p < 0.01, *** *p* < 0.001

## Discussion

Prior research has argued the importance of protecting patient privacy as an important step in implementing successful EMR in healthcare facilities [1], and a privacy policy should be designed to fulfill this goal. Through compliance with a stated privacy policy, employees of these healthcare facilities can effectively protect the privacy of the patients. Despite several studies which have investigated the determinants of adherence to a privacy policy [4, 5, 8, 11, 58], patient privacy and the ultimate success of EMR remain dependent on continuance protection rather than on one time protection. Surprisingly, prior to this particular study, little, if any, effort has been made to fill this gap in the literature.

The primary purposes of our study were to understand the drivers of continuance intention of privacy policy compliance from both motivational and habitual perspectives. We proposed that continuous intention is driven by a hospital staff’s habit formation, which in turn is motivated by satisfaction, self-efficacy, perceived usefulness, and facilitating conditions. Overall, we found that habit is a key element to positively predicting the continuous intention of hospital staff’s adherence to privacy policy, and habit can be formed through both extrinsic and intrinsic motivations. We identified three significant motivational antecedents of habit to be self-efficacy, perceived usefulness, and facilitating conditions. By comparing the importance of the three antecedents to habit development, facilitating conditions are seen to play a primary role, followed by self-efficacy, and then by perceived usefulness. Satisfaction, contrary to our expectation, was not considered as a significant antecedent of habit. Several academic and practical implications can be derived from our findings.

### Academic implications

Based on the reported findings, we would propose several points that might be worthy of consideration for further theory development. First, the literature [59] has considered continuance behavior (or, repeat behavior) to be guided by an individual’s cognitive process. Hence, much effort is devoted to explaining continuance intention directly from these perspectives, such as perceived usefulness, satisfaction, etc. However, as EMR becomes more prevalent among healthcare facilities, and thus healthcare professionals can access EMR anywhere and at any time without much involved cognitive process, habit may play a significantly larger role in protecting patient privacy. We therefore hope that our study will contribute towards future development of habit formation to ensure patient privacy protection in EMR usage.

Second, the significant antecedents of habit formation are found to include perceived usefulness, facilitating conditions, and self-efficacy; and, they are ranked in importance as follows: facilitating conditions, self-efficacy, and perceived usefulness. It implies that facilitating conditions are the most important determinant of habit formation. Prior literature [15, 22, 25, 60] asserts that intrinsic motivation overpowers extrinsic motivation. Interestingly, our study determined that extrinsic motivation (facilitating conditions) outweighs intrinsic motivation (self-efficacy). A possible explanation for such a finding may be due to the purposes of such behavioral intention being either egoistical or altruistic in nature. In our study, the ultimate purpose of adherence to privacy policy is to directly protect patients’ information, while prior studies [15, 22, 25] are primarily related to providing protection for themselves.

Third, habit cannot be determined by means of satisfaction, a finding that is not in line with prior evidence [23, 28, 29]. One plausible explanation for this occurrence might be that any existence of a privacy policy may impose significant burdens on hospital staff to maintain a compliant position, which is therefore less likely to positively form their habits. Further, compliance to stated privacy policy may not bring about expected rewards that would lead to the satisfaction of hospital staff as product or service consumption might evince [61].

Fourth, the inclusion of facilitating conditions and self-efficacy in our model has provided additional perspectives. Facilitating conditions and self-efficacy are usually considered before a behavior is conducted in terms of other behavioral theory [51]. The inclusion of these two variables in our model has allowed a broader picture of hospital staff’s perceptions in the post-behavioral phase, and it has created a richer understanding of their continuance intentions.

### Practical implications

The introduction of EMR may put hospital managers in situations where they must directly confront the important issue of patient privacy-protection. We hold that hospital managers may profit from knowing the influence of habit and the motivations of habit formation when encountering situations that call for continuance protection of EMR privacy. Knowing the influence of habit and the motivations of habit formation will not only force hospital managers to inspect whether or not to encourage the habitualization of EMR privacy-protection, but it will also help them to take proper actions for creating a more habit-conducive environment. First of all, we found that self-efficacy predicts the formation of a privacy-protection habit among hospital staffers. This finding may suggest that managers can launch EMR privacy-protection training programs to equip hospital staff with required knowledge, skills, or tools deemed necessary for their adherence to a stated privacy policy. Second, the significant result of perceived usefulness may also imply that the above privacy-protection training programs may focus on cultivating a perception that privacy policy is an effective means of protecting EMR privacy. Third, facilitating conditions is also a key to privacy-protection habit development. This finding may imply that hospital management should ensure all the required resources (i.e., hardware, software, and codified procedures) are in place for hospital staff to protect the privacy of patient and EMR facility. Further, any changes in EMR-related hardware, software, or codified procedures should not make hospital staff feel a huge difference has transpired. Otherwise, the formation of privacy-protection habit may not be immediately possible.

Several limitations should be noted in our study. First, the data was collected from only one Taiwanese medical center without comprising samples from other hospitals. Hence, the generalizability of our finding may in fact be limited. Further, in order to avoid any interruption of patient-care activities, the survey used a self-reporting method to investigate behavioral intention among staff rather than through direct observation or through the recording of participants’ actual compliance behavioral patterns. Future research can therefore examine the issue in order to better realize the associations among these investigated constructs examined as part of this study.

## Conclusions

By integrating both motivation and habitual perspectives, our study presented and then empirically verified a model used to examine continuous compliance with EMR privacy policies by hospital employees. Motivations including self-efficacy, perceived usefulness, and facilitating conditions are significant predictors of compliance habits; and, habits, in their turn, may significantly predict hospital staff’s continuance compliance intention. We also found that habit is a partial mediator between motivations and continuance compliance intention.

Our study findings may contribute to the body of knowledge in several ways. Firstly, our study adds to the existing literature of EMR privacy policy by harmonizing motivational and habitual perspectives, which may lay a theoretical foundation useful in investigating continuous compliance intentions to stated privacy policy. Second, our study proved that habit may also be regarded as a mediator of intrinsic and extrinsic motivations. Such a mediating effect of habit may yield a differing, nevertheless, useful perspective towards our understanding of continuous behaviors pertinent to overall privacy-policy adherence issues. Third, the findings of our study also proposed suggestions for health authorities and hospitals useful to foster effective strategies necessary to improve hospital staff’s continuous adherence to privacy policy in order to secure the overall safety of and responsible access to EMR.

## Appendix

**Table 6.** Questionnaire. The questionnaire used in this study

Constructs	Items	Source
Self-efficacy	I can comply with EMR privacy policy even if there is no one around to help me	Taylor and Todd [51]
	I can comply with EMR privacy policy if I have adequate time to complete my job	
	I can comply with EMR privacy policy using only manuals or documents for reference	
	I am confident in my ability to comply with EMR privacy policy	
Facilitating condition	I have access to the resources needed to comply with EMR privacy policy	Taylor and Todd [51]
	I can comply with EMR privacy policy whenever and however I want	
	I have full control over my compliance with EMR privacy policy	
Perceived usefulness	Compliance with EMR privacy policy is of benefit to me	Davis [36]
	The advantages of adherence to EMR privacy policy outweigh the disadvantages	
	Overall, compliance with EMR privacy policy is advantageous	
Satisfaction	I am satisfied with my decision to comply with EMR privacy policy	Bhattacherjee [50]
	My choice to compliance with EMR privacy policy was a wise one	
	I am happy with my earlier decision to comply with EMR privacy policy	
	My experience with adherence to EMR privacy policy was very satisfactory	
Habit	Compliance with EMR privacy policy has become automatic to me	Limayem and Cheung [52]
	Compliance with EMR privacy policy is natural to me	
	When taking care of patients, compliance with EMR privacy policy is an obvious requirement for me	
Continuous intention to comply with EMR privacy policy	I intend to continue complying with EMR privacy policy in the future.	Bhattacherjee [50]
	I will always try to comply with EMR privacy policy in my daily life.	
	I plan to continue to adhere with EMR privacy policy frequently.	

## Abbreviations

EMR: Electronic medical records
M: Mean
p: Probability value
PLS: Partial least squares
SD: Standard deviation
SE: Standard error
t: t statistics
β: Path coefficient
